# Micromagnetic Microstructure- and Stress-Independent Materials Characterization in Reactor Safety Research

**DOI:** 10.3390/ma14185258

**Published:** 2021-09-13

**Authors:** Cyril Zimmer, Yashashwini Nikhitha Rallabandi, Klaus Szielasko, Christian Eichheimer, Michael Luke, Sargon Youssef

**Affiliations:** 1 Fraunhofer Institute for Nondestructive Testing IZFP, Campus E3 1, 66123 Saarbrücken, Germany; yashashwini.rallabandi@izfp.fraunhofer.de (Y.N.R.); klaus.szielasko@izfp.fraunhofer.de (K.S.); sargon.youssef@izfp.fraunhofer.de (S.Y.); 2 Fraunhofer Institute for Mechanics of Materials IWM, Wöhlerstraße 11, 79108 Freiburg, Germany; christian.eichheimer@iwm.fraunhofer.de (C.E.); michael.luke@iwm.fraunhofer.de (M.L.)

**Keywords:** 3MA, micromagnetic materials characterization, machine learning, NDE, NDT, reactor safety research

## Abstract

Reactor safety research aims at the safe operation of nuclear power plants during their service life. In this respect, Fraunhofer IZFP’s micromagnetic multiparameter, microstructure, and stress analysis (3MA) has already made a significant contribution to the understanding of different aging mechanisms of component materials and their characterization. The basis of 3MA is the fact that microstructure and mechanical stress determine both the mechanical and magnetic material behavior. The correlation between features of magnetic and mechanical material behavior enables the micromagnetic prediction of mechanical properties and stress, both of which can decisively influence the service life. The Federal Ministry for Economic Affairs and Energy (BMWi) funded this research, handling the mutually superimposed microstructural and stress-dependent influences, a substantial challenge, especially under practical conditions. This superposition leads to ambiguities in the micromagnetic features. The 3MA testing system has been extended by more sophisticated evaluation methods being able to cope with more complex datasets. Investigations dealing with the expansion of the feature extraction and machine learning methods have led to a more precise distinction between microstructural and stress-dependent influences. This approach provides the basis for future applications in reactor safety.

## 1. Introduction

Despite the German phase-out from nuclear energy, the highest safety requirements for the operation of nuclear power plants during their remaining service life are still vital for all countries. The Federal Ministry for Economic Affairs and Energy (BMWi), as a funding body, therefore, supports research to maintain German expertise in the field of reactor safety.

Reactor pressure vessels (RPVs) are subject to different influences, such as neutron-degradation, plastic deformation, and temperature variations [1]. Although material conditions are regularly checked with charpy tests, these tests can only be repeated in limited numbers [1]. To address this issue, non-destructive testing methods (ndt-methods) are used to detect changes in material conditions to disburden destructive testing methods [1]. The micromagnetic multiparameter, microstructure, and stress analysis (3MA)-based testing systems of the Fraunhofer Institute for Nondestructive Testing (Fraunhofer IZFP) successfully contributed to the study of RPV-steel conditions such as copper precipitates and neutron induced embrittlement [2,3,4] and has proven its capabilities in several other fields of application, such as stress determination and general characterization of material inhomogeneities [5,6,7,8].

The main goal of this paper is the further distinction of microstructural and mechanical stress influences utilizing the 3MA-X8 testing system. As previous research has shown, the superposition of both influences has a non-negligible effect on micromagnetic methods on the inspection of RPV-steel [2]. The extent to which influence ambiguities can be reduced are examined and compared to former approaches.

The basis of 3MA is the fact that microstructure and mechanical stress determine both the mechanical and magnetic material behavior [9]. Therefore, correlations exist between mechanical and magnetic material behavior [9]. By using this correlation, the mechanical material properties can be predicted by acquiring magnetic features. The magnetic behavior can be characterized by features, which are extracted from the magnetic hysteresis [10]. Acquiring the magnetic hysteresis on components with a top-mounted sensor is usually infeasible because the direct measurement of the hysteresis involves special requirements, such as specific specimen geometries, coils comprising the specimen, and a low excitation frequency [11].

Fraunhofer IZFP’s 3MA-X8 testing system acquires magnetic features with a top-mounted sensor by combining three different micromagnetic ndt-methods to generate hysteresis-like features [1,12,13]. Recent research made use of an automated feature extraction, creating further micromagnetic features [12,13]. Machine learning methods extract the desired information out of the feature space to classify or quantify the acquired measurements [6].

Within the scope of this paper, the feature space was further extended by the raw signal data. Hence, depending on the measurement presets (sample rate, magnetization frequency, etc.), over a thousand features were generated. The focus of this research is on linear machine learning methods in combination with a high dimensional feature space. The extended feature space is compared to the original 21-dimensional feature space to determine possible improvements.

## 2. Materials and Methods

### 2.1. Materials

**Figure 1:**
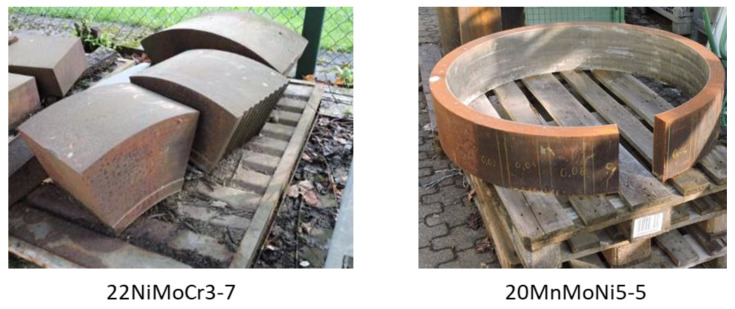
Reactor components provided by Fraunhofer IWM.

The materials used were 20MnMoNi5-5 and 22NiMoCr3-7. Both materials are in use as RPV-steel [14,15]. The specimens were cut out of reactor components shown in Figure 1.

The Fraunhofer Institute for Mechanics of Materials (Fraunhofer IWM) optimized the specimens’ shape for the different deformation procedures while still maintaining accessibility for a micromagnetic sensor.

### 2.2. Influences

**Figure 2:**
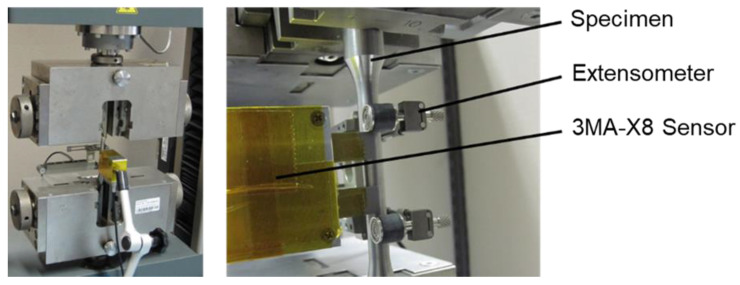
Measurement setup of a 3MA-X8 measurement of micromagnetic features during elastic tensile tests.

The examined dataset consisted of twelve different microstructures for classification and a continuous variation of mechanical stress for regression purposes. The microstructural states were adjusted beforehand. The micromagnetic measurements were conducted during elastic tensile tests as seen in Figure 2.

#### 2.2.1. Plastic Deformation

Fraunhofer IWM conducted mechanical tests introducing microstructural changes by plastic deformation [1,16]. For each material, six different microstructural states were adjusted by utilizing two different test methods with three different conditions. The methods used were plastic deformation introduced by tensile tests with strain levels of 0.8%, 2%, and 4% and low cycle fatigue tests (LCF-Tests) with 30%, 45%, and 60% of the determined lifetime. For each condition, three specimens were used.

#### 2.2.2. Elastic Stresses

Tensile tests continuously increasing the stress from 0% to 50% of the maximum yield strength of each material introduced elastic stresses during the in situ measurements. During the tests, the tensile stress was measured, synchronized, and saved to act as a reference value for the magnetic features. Prior to performing the measurements, the samples were demagnetized to minimize the influence of any residual magnetic fields.

### 2.3. Feature Engineering

**Figure 3:**
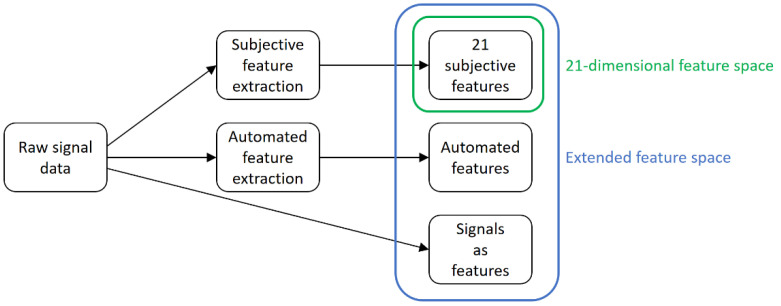
Feature engineering block diagram.

Different approaches are utilized to quantify changes in the magnetic behavior. These feature extraction methods result in three different categories of features. In addition to the 21 subjective features of the 3MA-X8 testing system [4,7], recent research has added automated features to the overall feature space [12,13]. In the course of this manuscript, the raw data samples expand the feature space even further. Figure 3 shows an overview of the different feature extraction methods and the definition of the 21-dimensional and the extended feature space via a block diagram. For excitation, two superimposed voltage controlled sinusoidal signals of different frequencies are applied to the 3MA-X8 sensor, which consists of a U-shaped electromagnet [12]. While the lower magnetization frequency (10–200 Hz) passes through operating points in the magnetic hysteresis, the higher eddy current frequency (500–5000 Hz) is used to perform an eddy current analysis at the respective points [13]. The resulting complex eddy current impedance signal, along with the raw signals of voltage and current, enter the different feature extraction methods. The measurements performed in this research were conducted with a magnetization signal of 3.5 V at 100 Hz and an eddy current signal of 1.5 V at 4 kHz. The incoming signals were sampled at 25 kHz. This parameterization results in 1247 features, which are divided into 21 subjective, 226 automated, and 1000 signal features.

#### 2.3.1. Subjective Feature Extraction

**Figure 4:**
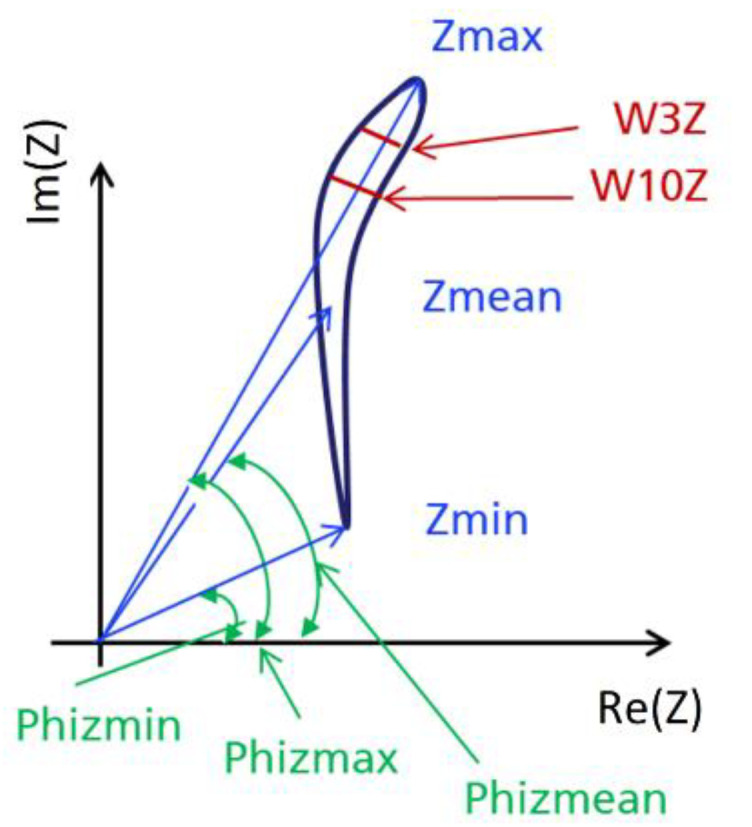
Schematic representation of the eddy current impedance loop [4].

The subjective feature extraction consists of three different testing methods with different sensitivities, the eddy current analysis, the incremental permeability analysis, and the harmonic analysis [13]. Each subjective feature is defined as a certain characteristic of the corresponding methods signal plot. Plotting the complex impedance over the real and imaginary axes creates the eddy current impedance loop [4]. This loop represents the impedance values for different operating points during a magnetization period of the hysteresis [4]. Figure 4 illustrates the eddy current impedance loop, as well as the feature extraction. Table 1 further describes the exact definition of the subjective features.

**Figure 5:**
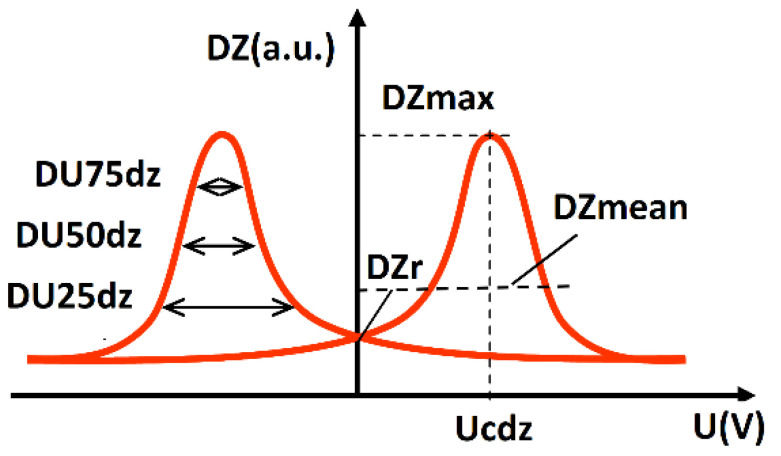
Schematic representation of the incremental permeability curve [4].

The incremental permeability curve is generated by plotting the change in coil impedance over the magnetization voltage [4]. This plot provides information about the permeability as a function of the magnetization [4]. Figure 5 illustrates the incremental permeability curve, as well as the feature extraction. Table 2 further describes the exact definition of the subjective features.

**Figure 6:**
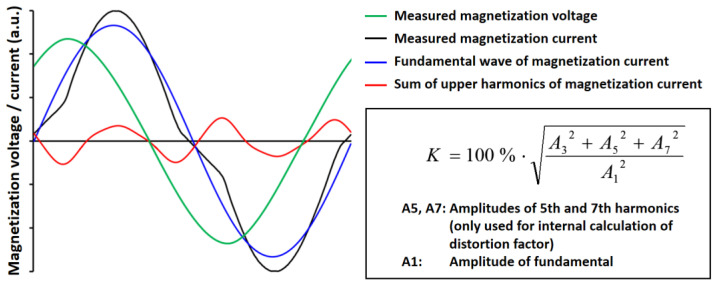
Schematic representation of the harmonic analysis [4].

The nonlinearities of the hysteresis cause the distortion of the magnetization current [4]. This nonlinear behavior is quantified using a fast Fourier transformation (FFT) [4]. The resulting FFT provides the foundation for the features of the harmonic analysis [4]. Figure 6 illustrates the harmonic analysis, as well as the feature extraction and feature calculations. Table 3 further describes the exact definition of the subjective features.

#### 2.3.2. Automated Feature Extraction

The automated feature extraction is a systematic approach to generalize the procedures of the subjective feature extraction [12]. This more sophisticated evaluation of all available time signals leads to a more sensitive representation of the magnetic behavior [12,13]. The FFT is analyzed up to the tenth harmonic with magnitude and phase [13]. Characteristic points of all signals (e.g., maximum, minimum, zero points) as well as values of other signals at the aforementioned points contribute to the feature extraction [12].

#### 2.3.3. Signals as Features

Every sample of the raw signals is added to the feature space. The raw signals include the measured voltage, current, and the real and imaginary part of the complex impedance.

### 2.4. Machine Learning Methods

Different machine learning algorithms are used to cope with the difficulties occurring in a multidimensional feature space. In contrast to prior research that focused on algorithms with hyperparameter optimization, the approach in this paper is based on a more complex feature space and linear algorithms without the need of hyperparameter optimization [12]. An additional advantage of linear algorithms is the tendency to be less prone to overfitting, which makes them a reliable choice, especially in practical use cases [7].

#### 2.4.1. LDA

The machine learning algorithm linear discriminant analysis (LDA) was applied to the feature space to extract the essential information content for material and property analysis. LDA is a supervised method used in machine learning [17]. With the help of determined discriminant functions, a coordinate transformation with subsequent linear projection onto new secondary characteristics (canonical variables) was carried out. Since this is a supervised procedure, the class memberships of the training data in the feature space are known. The rotation of the coordinate system is performed according to the principle of minimizing the variance within a class (intragroup variance) and maximizing the variance between classes (intergroup variance). The number of classes to be separated is crucial for the number of discriminant functions. To separate n classes, a maximum of n−1 discriminant functions can be determined. The information content of the classes to be separated is highest in the first discriminant function and decreases continuously.

#### 2.4.2. KNN-Classifier

The classification is based on the machine learning algorithm k-nearest neighbor (kNN) classifier [17]. When performing the kNN-classification, the data to be classified are compared to the trained data. For this purpose, the Euclidean distances of the new measured value to each trained data point are determined, and then sorted according to their distance. In the k smallest distances, the predicted class is determined. The most frequently occurring class in the k smallest distances is the predicted class. For classification problems, the class memberships are known in training. An evaluation of the model quality is only possible on a referenced data set. Hence, without knowledge of the target variable to be classified, it is not possible to evaluate whether the classification was correct.

## 3. Results

### 3.1. Preliminary Examinations

**Figure 7:**
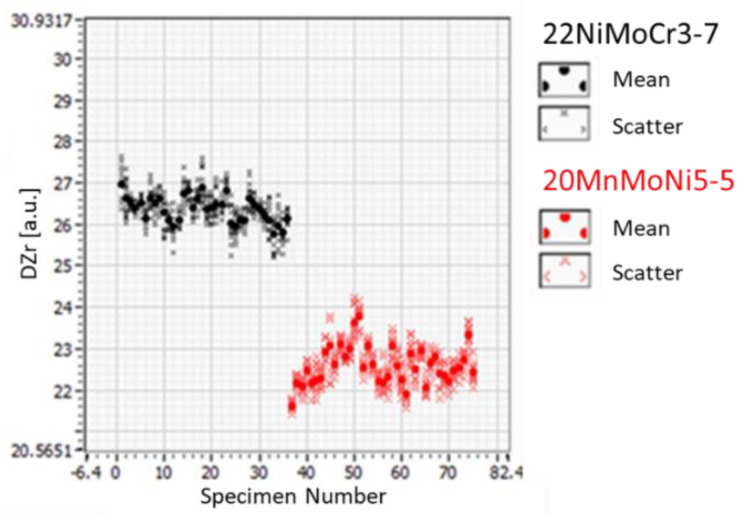
Preliminary micromagnetic measurements on all specimens, feature DZr.

After the specimens were machined from the reactor components, a preliminary micromagnetic test was carried out to detect significant outliers in them. The feature DZr of the subjective feature extraction was chosen to evaluate the micromagnetic test. DZr represents the incremental permeability at remanence [4]. As shown in Figure 7, aside from the normal variation of the specimen measurements, no significant outliers were detected.

**Figure 8:**
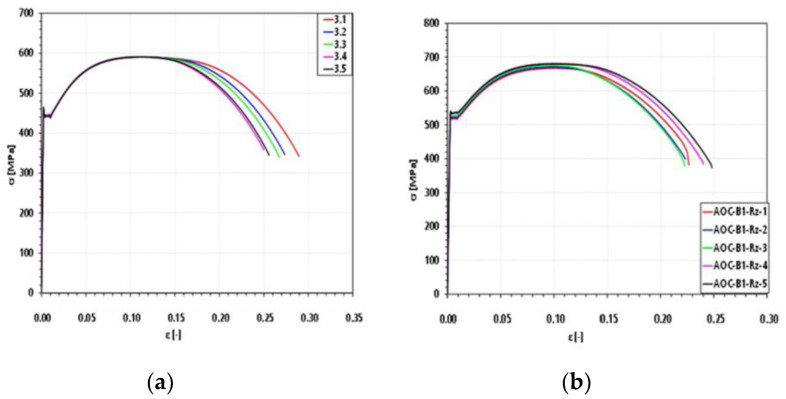
Stress-strain curves for each material during preliminary examinations: (**a**) 20MnMoNi5-5 and (**b**) 22NiMoCr3-7.

Tensile tests were performed to determine and characterize the basic material properties. A low variation of yield strength for each material ensures sufficient specimen homogeneity. Based on these tests, the strain levels (plastic deformation) 0.8%, 2%, and 4% were chosen to represent different material states. Figure 8 shows each of five tensile tests per material. Each stress-strain curve corresponds to a specimen of the respective material. All specimens were in the same condition for statistical verification. The legend represents the specimen identifier. The final elastic tensile tests for acquiring the dataset were designed based on 50% of the yield strength for each material.

LCF-tests determined the fatigue limit defined by the number of cycles to failure. A strain amplitude of 0.35% was applied to all specimens at a load ratio of 0.1. The obtained cycles to failure and their standard deviation are shown in Table 4. Due to a high standard deviation of up to ~20%, relative to the cycles to failure, the number of applied cycles for the main experiment was highly limited towards the maximum cycles to failure. As a tradeoff between maintaining sufficient microstructural change and protecting the specimens from accidental breakage during testing, 30%, 45%, and 60% of the averaged cycles to failure were chosen.

**Figure 9:**
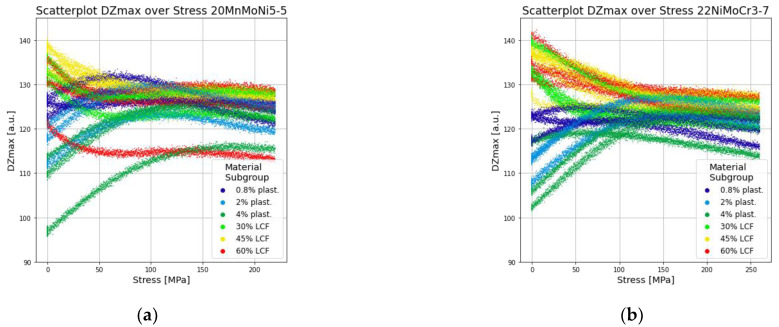
Stress-dependent scatterplot of feature Dzmax: (**a**) 20MnMoNi5-5 and (**b**) 22NiMoCr3-7.

In order to obtain a rough overview of the stress and microstructure dependencies, Figure 9 shows the feature DZmax as an example of one of the 21 subjective features in dependence of both microstructure and stress influences. The scatterplot displays no clear overall interrelationship. An ambiguity regarding the stress and microstructure dependency is present. Therefore, neither stress-independent microstructure characterization nor microstructure-independent stress determination is possible with this single feature.

### 3.2. Stress-Independent Microstructure Characterization

In prior to the evaluation, the dataset, containing 103,884 samples, was randomly split into a test and train dataset with a ratio of 20% test data to 80% train data. Training and testing with different datasets can reveal overfitting when the training dataset is significantly better predicted than the test dataset [18]. The classification was carried out in two steps. First, the LDA transformed the high dimensional feature space into four canonical components for a more intuitive grasp of the overall distribution and distinguishability. The kNN classifier predicted the classes based on the LDA output. The classification results are shown in a confusion matrix. The resulting misclassification rate represents the overall performance of the classification and enables a quantifiable comparison of different approaches. The two different approaches used consist of two individual feature spaces, the original 21-dimensional feature space on one hand and the extended feature space with additional automatic feature extraction as well as the raw signal data on the other hand.

**Figure 10:**
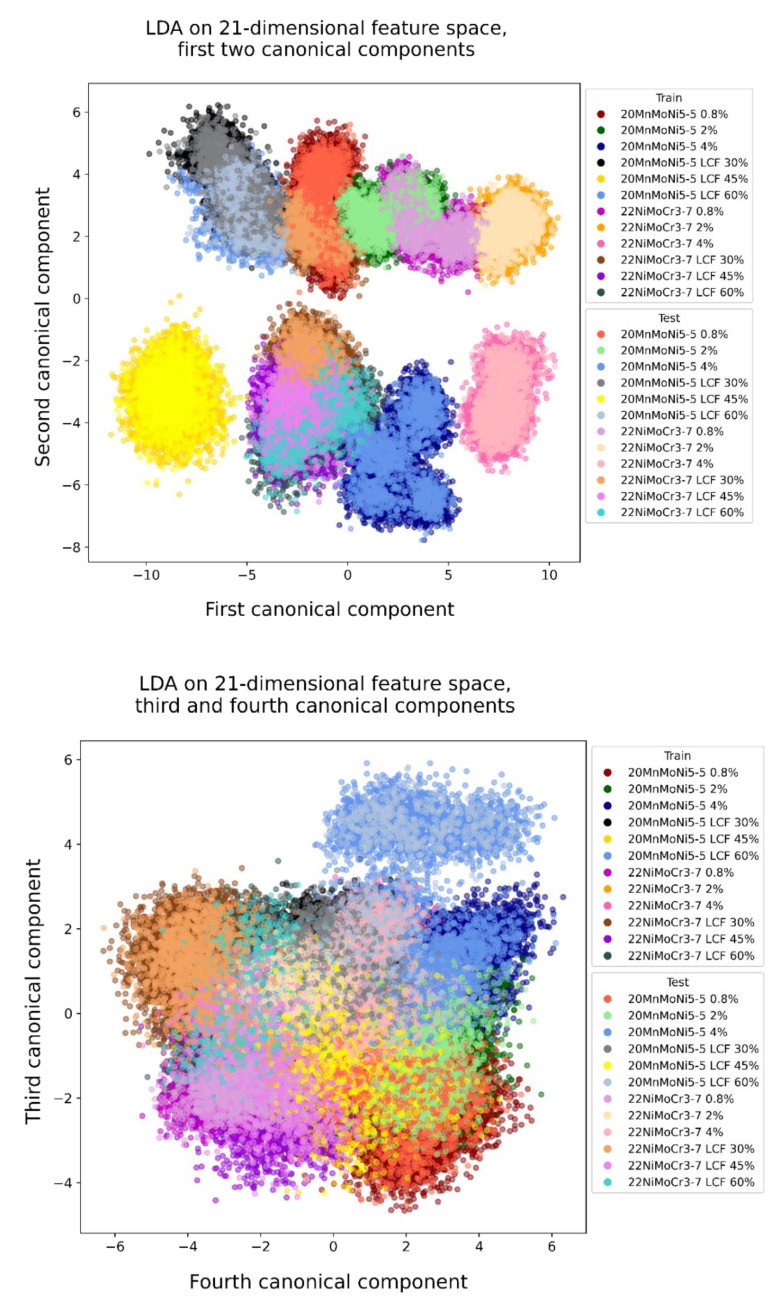
Stress-independent microstructure characterization of the first four canonical components transformed by LDA, 21-dimensional feature space.

**Figure 11:**
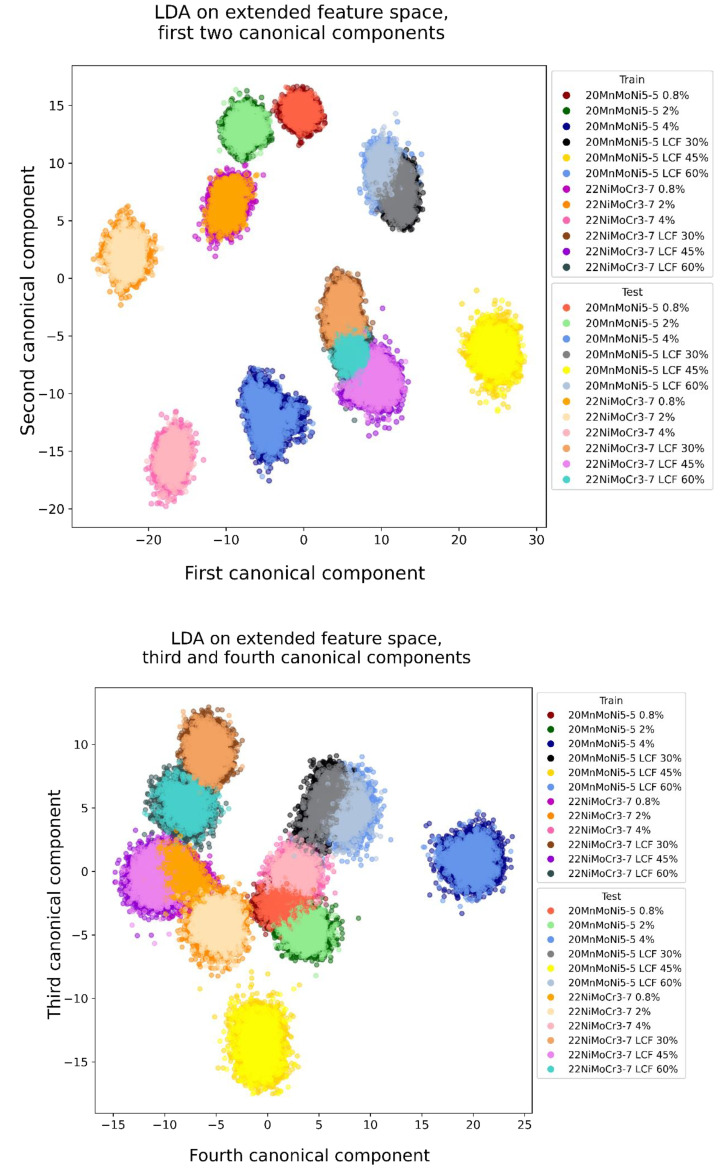
Stress-independent microstructure characterization of the first four canonical components transformed by LDA, extended feature space.

The first step, transforming the data with a LDA of the 21-dimensional feature space, is shown in Figure 10 and the transformed data of the extended feature space is shown in Figure 11. The first four canonical components are presented as two-dimensional plots to illustrate the group-based clustering the LDA generated. The legend of each diagram shows the colors of the test data while the train data are shown as the same color with a lighter shade.

The first subjective impression is an improvement in the distinction of each of the classes by feature space extension. The clusters of the 21-dimensional feature space show much more overlap than the clusters of the extended feature space. In both cases, the transformation of the test data does not vary significantly from the training data. Therefore, a low risk of overfitting can be assumed.

**Figure 12:**
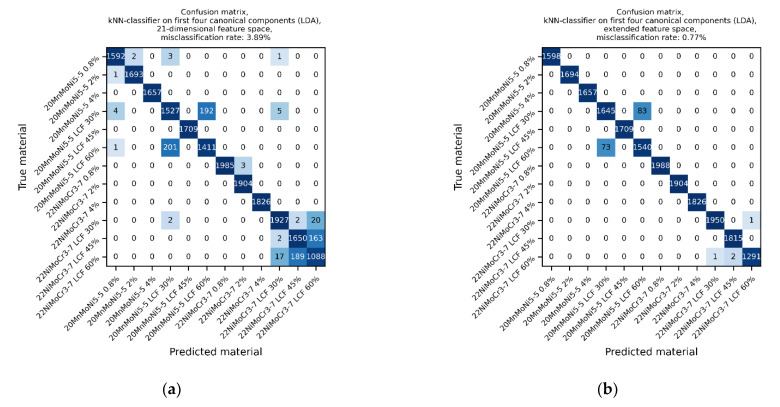
Confusion matrices of kNN-classification based on LDA transformation: (**a**) 21-dimensional feature space and (**b**) extended feature space.

The kNN-classification was performed solely on the test data to survey the prediction of unknown data. Only the first four canonical components of each feature space determined the space, in which the kNN-classifier predicted the classes. The kNN-classifier was parametrized to predict the class with k = 5 neighbors and a uniform weighting. The confusion matrices were used to determine the misclassification rate. The result of each feature space is shown in Figure 12. Comparing the misclassification rate of both feature spaces, the extended feature space misclassified with 0.77% about four times less often than the 21-dimensional feature space with 3.89%.

### 3.3. Microstructure-Independent Stress Determination

**Figure 13:**
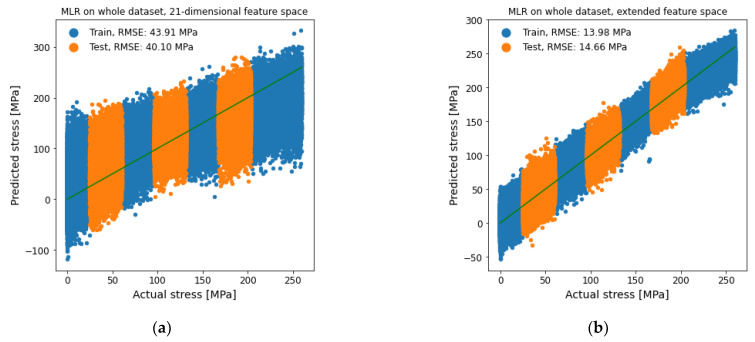
MLR for stress determination on whole dataset. (**a**) 21-dimensional feature space and (**b**) extended feature space.

The dataset on which the stress determination was carried out is identical to the dataset of the prior material determination. Before using a multiple linear regression (MLR), the datasets of each feature space were divided into range-based subgroups to detect overfitting and overgeneralization. For each individual dataset, a total of seven subgroups with an equal number of samples were used. This sample range-based subdivision was chosen to determine a fixed ratio of 3/7 testing and 4/7 training data. The alternating assignment to testing and training data enabled us to see how well the model would interpolate the test data. In addition to the typical regression on the whole dataset, an additional approach based on hierarchical modeling was investigated. Here, the classification first estimated a microstructure subgroup (e.g., material) before using a subgroup-specific regression model. This approach required a sufficient classification, as well as an increasing prediction quality when using a subgroup-specific regression model. Material- and influence-based subgroups were analyzed to see, how much different subgroup specific regression models improved the stress prediction quality. Figure 13 compares the 21-dimensional feature space with the extended feature space to give an insight over the improvements. A comparable training and testing root mean square error (RMSE) in each of the models indicates that overfitting did not occur. Comparing the test RMSE of the different feature spaces, the extended feature space performed substantially better than the 21-dimensional feature space. The test RMSE of 14.66 MPa of the extended feature space was about 37% of the 21-dimensional feature space with 40.10 Mpa.

**Figure 14:**
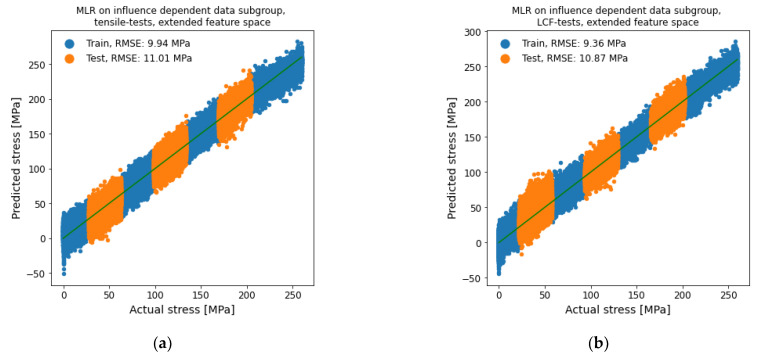
MLR for stress determination on influence-dependent data subgroup with extended feature space. (**a**) plastification via tensile tests and (**b**) LCF-tests.

The first subgroup division shown in Figure 14 was carried out with an influence-based division of the dataset. Both datasets were trained with the extended feature space. Both divisions performed similar with a test RMSE of 11.01 MPa for the plastification via tensile tests and 10.87 Mpa for the LCF-tests. The test RMSE was lowered to about 76% compared to the regression on the whole dataset with the extended feature space. Therefore, the hierarchical modeling was able to get a significantly better result by using an influence-based subgroup division.

**Figure 15:**
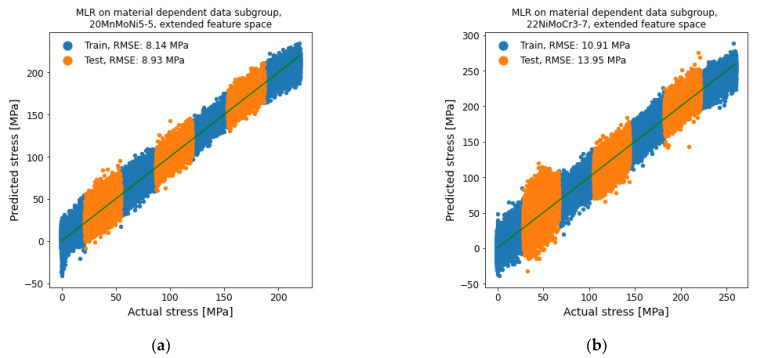
MLR for stress determination on material-dependent data subgroup with extended feature space. (**a**) 20MnMoNi5-5 and (**b**) 22NiMoCr3-7.

The second subgroup division based on the different materials is shown in Figure 15. The datasets were trained with the extended feature space. The test RMSE of each material subgroup differed clearly, in contrast to the prior influence-based subgroup division where the resulting RMSE of each subgroup was similar. For the material 20MnMoNi5-5, the RMSE was remarkably reduced to about 61% with 8.93 MPa compared to the regression on the whole dataset with the extended feature space. The RMSE for the material 22NiMoCr3-7 did not change considerably with 13.95 Mpa to only 95% of the regression on the whole dataset with the extended feature space. The material-based subgroup division therefore improved the prediction quality of the material 20MnMoNi5-5 distinctly more than for the material 22NiMoCr3-7.

## 4. Discussion

The approaches applied in the scope of this publication have been proven suitable for the distinction of microstructural and mechanical stress influences. In contrast to prior research, using an extended feature space containing an automatic feature extraction as well as raw signals in combination with linear machine learning methods reduced the need for hyperparameter optimization. By evading the hyperparameter optimization, the risk of overfitting by improperly parametrized models was avoided. However, general overfitting still has to be investigated with separate testing and training datasets to verify the models’ prediction quality. For both regression and classification models, the extended feature space significantly improved the prediction quality compared to the original 21-dimensional feature space. The great advantage of the extended feature space is that the subjectively imperceptible signal properties are also included in the evaluation [12]. Hierarchical models improve the prediction quality further for regression models by first reducing complexity in known influences before focusing on predicting certain influences. Nevertheless, each subgroup specific model has to be surveyed to avoid overfitting and based on the quality of the results, it has to be decided, whether a hierarchical model is appropriate for the application.

This research creates a solid foundation for future practical implementation in reactor safety. Various other 3MA-X8 applications can also be improved with the approach investigated in this research, especially in similar conditions where superimposed influences result in complex datasets.

## Figures and Tables

**Table 1:** Subjective features of the eddy current analysis.

Feature	Description
Zmean	Average of absolute impedance of impedance loop
Zmin	Minimum of absolute impedance of impedance loop
Zmax	Maximum of absolute impedance of impedance loop
Phizmean	Phase of the average impedance
Phizmin	Phase of the minimum impedance
Phizmax	Phase of the maximum impedance
W3Z	Width of the impedance loop at 3% of maximum
W10Z	Width of the impedance loop at 10% of maximum

**Table 2:** Subjective features of incremental permeability analysis.

Feature	Description
DZmax	Maximum value of incremental permeability
DZmean	Average value of incremental permeability
DZr	Value of incremental permeability at U = 0
Ucdz	Voltage at maximum of incremental permeability
DU75dz	Width of incremental permeability at 75% of maximum
DU50dz	Width of incremental permeability at 50% of maximum
DU25dz	Width of incremental permeability at 25% of maximum
Rem	Voltage offset of DZmax

**Table 3:** Subjective features of the harmonic analysis.

Feature	Description
Vmag	Amplitude of magnetization voltage
Imag	Amplitude of magnetization current
K	Distortion factor
A3	Amplitude of third harmonic
P3	Phase shift of third harmonics

**Table 4:** Results of Preliminary LCF-tests.

Material	Cycles to Failure	Standard Deviation
20MnMoNi5-5	3238	432 (13.34%)
22NiMoCr3-7	6020	1322 (21.96%)

## Data Availability

The data presented in this study are available on request from the corresponding author.

## References

[1] Dobmann G. (2006). NDE for material characterisation of ageing due to thermal embrittlement, fatigue and neutron degradation. IJMPT.

[2] Altpeter I., Dobmann G., Szielasko K. Detection of copper precipitates in 15 NiCuMoNb5 (WB 36) steel by using micromagnetic NDE techniques. Proceedings of the 3rd International Conference on NDE in Relation to Structural Integrity for Nuclear and Pressurised Components.

[3] Altpeter I., Dobmann G., Hübschen G., Kopp M., Tschuncky R. Nondestructive characterization of neutron induced embrittlement in nuclear pressure vessel steel microstructure by using electromagnetic testing. Proceedings of the 8th International Conference on NDE in Relation to Structural Integrity for Nuclear and Pressurized Components.

[4] Rabung M., Kopp M., Gasparics A., Vértesy G., Szenthe I., Uytdenhouwen I., Szielasko K. (2021). Micromagnetic characterization of operation-induced damage in charpy specimens of RPV steels. Appl. Sci..

[5] Rabung M., Amiri M., Becker M.M., Kopp M., Tschuncky R., Veile I., Weber F., Weikert-Müller M., Szielasko K., Casavola C. (2020). nondestructive characterization of Residual Stresses Using Micromagnetic and Ultrasonic Techniques. New Challenges in Residual Stress Measurements and Evaluation.

[6] Dobmann G., Boller C., Herrmann H.G., Altpeter I. Electromagnetic NDT for lifetime management by monitoring of ageingphenomena. Proceedings of the 12th International Conference of the Slovenian Society for NDT.

[7] Youssef S., Amiri M., Ballman H., Molenda D., Tschuncky R., Youssef A. Detection of hard spots and other material inhomogeneities on steel plates. Proceedings of the 12th ECNDT.

[8] Kurz J.H., Szielasko K., Tschuncky R. Non-destructive stress determination of steel elements in pre-stressed constructions using micromagnetic and ultrasound methods. Proceedings of the NDTCE.

[9] Cullity B.D., Graham B. (2008). Introduction to Magnetic Materials.

[10] Bertotti G. (1998). Hysteresis in Magnetism.

[11] Kneller E. (1962). Ferromagnetism.

[12] Youssef S., Zimmer C., Szielasko K., Schütze A. (2019). Automatic feature extraction of periodic time signals using 3MA-X8 method. TM-Tech. Mess..

[13] Szielasko K., Wolter B., Tschuncky R., Youssef S. (2019). Micromagnetic materials characterization using machine learning—Progress in nondestructive prediction of mechanical properties of steel and iron. TM-Tech. Mess..

[14] Sarkar A., Kumawat B.K., Chakravartty J.K. (2015). Ratchetting behavior of 20MnMoNi55 reactor pressure vessel steel. J. Nucl. Mater..

[15] Balart M.J., Knott J.F. (2008). Low temperature fracture properties of DIN 22NiMoCr37 steel in fine-grained bainite and coarse-grained tempered embrittled martensite microstructures. Eng. Fract. Mech..

[16] Korzekwa D.A., Matlock D.K., Krauss G. (1984). Dislocation substructure as a function of strain in a dual-phase steel. Metall. Mater. Trans. A.

[17] Pedregosa F., Varoquaux G., Gramfort A., Michel V., Thirion B., Grisel O., Blondel M., Prettenhofer P., Weiss R., Dubourg V. (2011). Scikit-learn: Machine learning in Python. JMLR 12.

[18] Müller A.C., Guido S. (2016). Introduction to Machine Learning with Python: A Guide for Data Scientists.

